# Assigning shark fin origin using species distribution models needs a reality check

**DOI:** 10.1098/rsbl.2020.0907

**Published:** 2021-07-14

**Authors:** Vincent Raoult, Michael I. Grant, Ana Paula Barbosa Martins, Leonardo Manir Feitosa, Matias Braccini, Diego Cardeñosa, John Carlson, Andrew Chin, Tobey Curtis, Luís Fernando Carvalho Costa, Luís Fernando Rodrigues Filho, Tommaso Giarrizzo, Jorge Luiz S. Nunes, João Bráullio L. Sales, Jane E. Williamson, Colin A. Simpfendorfer

**Affiliations:** ^1^ School of Environmental and Life Sciences, University of Newcastle, Ourimbah, New South Wales 2258, Australia; ^2^ Centre for Sustainable Tropical Fisheries and Aquaculture and College of Science and Engineering, James Cook University, 1 James Cook Drive, Townsville, Queensland 4811, Australia; ^3^ Integrated Fisheries Laboratory, Dalhousie University, Halifax, Nova Scotia, Canada B3H 4R2; ^4^ Bren School of Environmental Science and Management, University of California, Santa Barbara, CA 931117, USA; ^5^ Western Australian Fisheries and Marine Research Laboratories, Department of Primary Industries and Regional Development, Government of Western Australia, PO Box 20, North Beach, Western Australia 6920, Australia; ^6^ Department of Biological Sciences, Florida International University, 3000 NE 151st Street, North Miami, FL 33181, USA; ^7^ NOAA Fisheries Service, Southeast Fisheries Science Center, Panama City, FL 32408, USA; ^8^ Atlantic Highly Migratory Species Management Division, National Oceanic and Atmospheric Administration, National Marine Fisheries Service, Gloucester, MA 01930, USA; ^9^ Departamento de Biologia, Universidade Federal do Maranhão, Avenida dos Portugueses 1966, CEP 65080-805 São Luís, MA, Brazil; ^10^ Universidade Federal Rural da Amazônia (UFRA), Campus Universitário de Capanema, Rua João Pessoa 121, CEP 68700-030 Capanema, PA, Brazil; ^11^ Núcleo de Ecologia Aquática e Pesca da Amazônia, Universidade Federal do Pará, Avenida Perimetral 2651, Terra Firme, CEP 66040-170 Belém, PA, Brazil; ^12^ Departamento de Oceanografia e Limnologia, Universidade Federal do Maranhão, Avenida dos Portugueses 1966, CEP 65080-805 São Luís, MA, Brazil; ^13^ Grupo de Investigação Biologica Integrada (GIBI), Universidade Federal do Pará, Avenida Perimetral da Ciência, Km01, PCT-Guamá, Terreno 11, CEP 66075-750 Belém, PA, Brazil; ^14^ Department of Biological Sciences, Macquarie University, Sydney, New South Wales 2109, Australia

The conservation and management of shark populations have become urgent issues to ensure the future health of our oceans [1]. There are many drivers of the decline of shark populations, with the demand for shark fins being one of the more important [2]. Understanding fin origin can help identify regions for improved management, and hence has been the focus of recent research (e.g. Fields *et al*. [3], Cardeñosa *et al*. [4]). In a recent *Biology Letters* article, Van Houtan *et al*. [5] contributed to this work using data on species composition of shark fins at four markets and species distribution models (SDMs) to predict the probability of fin origin. Their purpose was to address knowledge gaps in source and trade routes of shark products, which currently limit the effective allocation of management resources. While the broad concept behind their paper is novel, we disagree with the results and conclusions owing to flaws in methodology and interpretation.

We fundamentally disagree with the central assumption of the paper that there is a direct link between species distribution and shark fin origin. This assumption relies on fisheries catch being equal through the distribution of a species, which we know is not true. Fishing effort that catches sharks is spatially heterogeneous [6] because of the patchy nature of target species and spatially explicit management arrangements (e.g. marine protected areas, shark sanctuaries, catch and effort limits). The fact that the size of a nation's exclusive economic zone accounted for more of the variation in Van Houtan *et al*.'s [5] estimate of a nation's contribution to the fin trade (*r^2^* = 0.48) than its elasmobranch catch as reported to FAO (*r^2^* = 0.20) underlines this erroneous assumption. An example of the dissonance caused by excluding fishing activity is northwestern Australia, where Van Houtan *et al*. [5] indicate a high probability of shark fin origin for many species, despite the area being closed to commercial shark fishing since 1993, and no operational fisheries to support suggested catch [7]. Such discrepancies have overinflated the estimated contribution of shark fins from nations as these factors have not been accounted for, leading to unrealistic conclusions about the source of fins in trade.

The paper's use of DNA data from some markets may be misleading since it assumed that all markets contributed equally to the global fin trade. For example, Feitosa *et al*. [8] collected samples from shark trunks (not fins) caught in waters of northern Brazil. These sources were not appropriate for global fin trade assessment as (i) there has been a shift in the supply chain from fins to meat in the area since 2010 [9], and (ii) unlike markets that aggregate samples from many nations, these samples only represented species from a single nation and so should not have been distributed to all waters where those species are known to occur. The paper also implicitly assumes that the proportion of fins in the four DNA studies relates directly to true global catches, and thus falsely deduces that species not found in these papers—like spiny dogfish *Squalus acanthias*—do not occur in the fin trade [4].

Many of the SDMs used by Van Houtan *et al*. were seriously flawed, with 21 of the 57 (more than 30%) having serious inaccuracies. In all flawed cases, the SDMs indicate species occurrence well outside their established geographical distributions known from decades of fishery and research data, which are reported in widely available species guides (e.g. [10,11]). These include SDM ‘habitat’ outside known latitudinal distribution, overlooked pelagic distributions and presence in oceans where they do not occur (figure 1). For example, mako sharks (*Isurus oxyrinchus*) primarily occur in the open ocean rather than coastal environments, grey reef sharks (*Carcharhinus amblyrhynchos*) do not occur in the Atlantic Ocean, smalleye hammerhead (*Sphyrna tude*s) occurs only in eastern South America (not globally) and great hammerheads (*Sphyrna mokarran*) are mostly coastal and not present to the latitudinal extent as suggested by the SDMs*.* This lack of a validity check against known distributions results in the allocation of species to exclusive economic zones (EEZs) in which they do not occur and hence erroneous probabilities of contributions to the fin trade. With more than 30% of SDMs having major flaws, the errors introduced to the estimation of the probability of fin origin are large.

**Figure 1.**
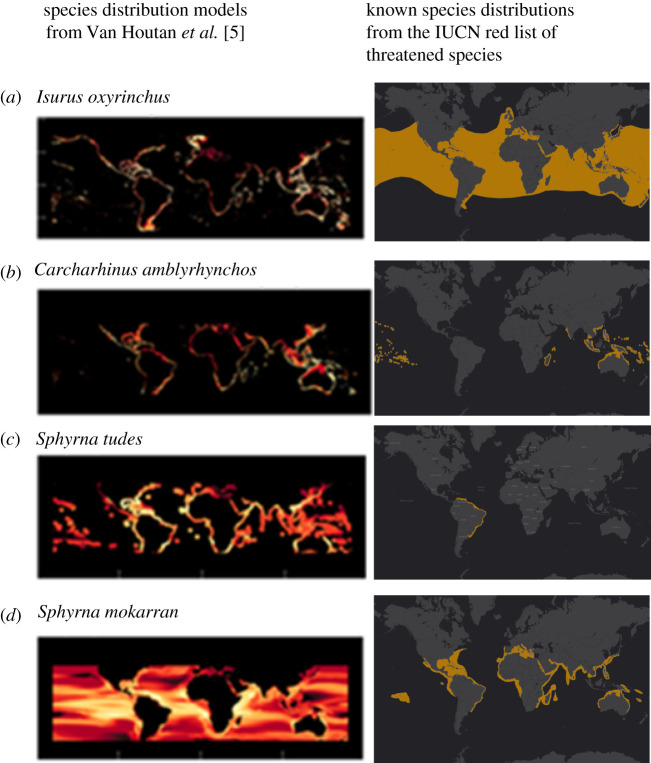
Comparison of SDMs from Van Houtan *et al*. [5] and known species distributions. Examples of (*a*) lack of pelagic distribution, (*b*,*c*) occurrence in ocean basins outside of their currently reported distribution and (*d*) occurrence is pelagic areas and outside of currently reported latitudinal distribution. Known species distributions from www.redlist.org. SDM-based maps from Van Houtan *et al*. [5] supplementary material.

The flaws in the methods used by Van Houtan *et al*. [5] mean that the conclusions that they have drawn are erroneous. First, we argue that their results at best show the probability of where species in the fin trade occur, not probabilities that ‘represent the top nations contributing the most shark fins to the global market’ (caption for figure 2 in Van Houtan *et al*. [5]). For example, the conclusion that Australia is the top contributor to the fin trade is impossible given that national shark and ray catch is less than 5000 t yr^−1^ [12], a level that cannot produce sufficient fins (they account for about 5% of landed weight, approx. 250 t) to account for it being the country supplying the most fins to the trade [13]. Their findings also contrast previous accounts of trade [10], and genetic evidence suggesting primarily Eastern Pacific origins for pelagic thresher sharks (*Alopias pelagicus*) and scalloped hammerhead sharks (*Sphyrna lewini*) and Indo-Pacific origins for silky sharks (*Carcharhinus falciformis*) [3,4]. These genetic tracking approaches provide more relevant outcomes for identifying the most prevalent source regions and important supply chain starting points for shark fins, and to prioritize conservation measures to these key regions.

If the authors had considered known locations of global fishing activity, the open ocean would appear a more likely origin for fins [14,15]. This flaw is best illustrated by blue shark *Prionace glauca*, bigeye thresher *Alopias supercilious* and shortfin mako shark, *Isurus oxyrinchus,* which together account for most (more than 50%) shark fins found in the market samples used to populate SDMs. These are all pelagic species and open ocean fisheries should have higher dominance in the origin probabilities. However, this result was not apparent because inaccurate SDMs and omission of relevant fisheries data created an unrealistic scenario of global shark fisheries. For example, considering available data on these three species, less than 1000 unprocessed t yr^−1^ (only a small portion of which are fins) are caught in Australia and the USA [16], and less than 3000 t yr^−1^ in Brazil [17]. If the authors had compared their results to known levels of national catch, their unrealistic results would have been highlighted. This omission means that the conclusion that coastal sharks supply the greatest part of the global fin trade is erroneous. Their conclusion is further complicated by never defining what a coastal species is—we suspect they mean species taken within EEZs (first line of Results and Discussion). If this is what they mean, then this is seemingly arbitrary compared to what is normally considered coastal. Typically, coastal species occur primarily on continental shelves, or close to shore where shelves do not exist [2].

The misinterpretations and methodological issues of the paper have resulted in inappropriate management recommendations for nations that are examples of best practice shark fisheries (e.g. USA and Australia; see Simpfendorfer & Dulvy [18]). Their conclusion of a ‘serial shift’ in shark fisheries to inshore waters contrasts with established trends (that have used time-series fisheries data) of fishing moving further offshore and into deeper waters [19]. This advice diverts attention away from the primary habitat (i.e. open ocean, not continental shelf) of the key taxa (e.g. blue and mako sharks) implicit in the shark fin trade. This could divert global management efforts away from open ocean fisheries and worsen conservation outlooks for open ocean species where conservation concern is high [20].

We do not question the occurrence of coastal shark species in the global fin trade, nor that opportunities exist to improve shark conservation within EEZs of numerous countries. However, prioritization of shark conservation efforts across countries and the high seas must consider the realities of the present distribution of species and fisheries activity, and existing national and international management arrangements.

## Data accessibility

This article has no additional data.
